# Honey vs. silver sulphadiazine

**DOI:** 10.4103/0970-0358.63947

**Published:** 2010

**Authors:** Viroj Wiwanitkit

**Affiliations:** Wiwanitkit House, Bangkhae, Bangkok, Thailand

Sir,

I read the recent publication by Baghel *et al*. on wound healing in burn patients with great interest.[1] Bahgel *et al*. concluded that "Honey dressing improves wound healing, makes the wound sterile in lesser time, has a better outcome in terms of prevention of hypertrophic scarring and post-burn contractures, and decreases the need of debridement irrespective of time of admission, when compared to SSD dressing".[1] Indeed, several reports confirm the possibility of using honey dressing for burn patients.[2,3] However, according to the meta-analysis, it is shown that "Honey dressings as an adjuvant to compression do not significantly increase leg ulcer healing at 12 weeks. There is insufficient evidence to guide clinical practice in other areas".[3] The main flaw of the report by Baghel *et al*. is the lack of a matched control group in the study.[1] In addition, an important problem to be concerned is the sterility of the honey. The contamination of Clostridium should not be overlooked.[4]
